# Dysphagia and associated factors among patients with acute ischemic stroke in Vietnam

**DOI:** 10.1016/j.amsu.2022.104887

**Published:** 2022-11-16

**Authors:** Nguyen Thi Thu Hien, Tran Huu Thong, Le Thanh Tung, Vo Hong Khoi, Dao Thi Thu Hoai, Tran Thi Tinh, Nguyen Van Huy, Vu Duy Kien

**Affiliations:** a Center of Neurology, Bach Mai Hospital, No.78 Giai Phong Street, Hanoi, Viet Nam; b Center of Emergency (A9), Bach Mai Hospital, No.78 Giai Phong Street, Hanoi, Viet Nam; c Nam Dinh University of Nursing, No.257 Han Thuyen Street, Nam Dinh, Viet Nam; d Department of Neurology, Hanoi Medical University, Hanoi, Viet Nam; e Department of Neurology, University of Medicine and Pharmacy, Vietnam National University, Hanoi, Viet Nam; f Clinical Nutrition Center, Bach Mai Hospital, No.78 Giai Phong Street, Hanoi, Viet Nam; g Health Innovation and Transformation Centre, Federation University, Victoria, Australia; h School of Medicine and Dentistry, Griffith University, Australia; i OnCare Medical Technology Company Limited, Hanoi, No. 77/508 Lang Street, Viet Nam

**Keywords:** Dysphagia, Prevalence, Acute ischemic stroke, Swallowing, Associated factors, Vietnam

## Abstract

**Background:**

Dysphagia is considered an important issue in managing and treating acute stroke, but there are currently no studies investigating this issue, especially for patients with acute ischemic stroke in Vietnam. Thus, we conducted this study to determine the prevalence of dysphagia and associated factors of dysphagia in patients with acute ischemic stroke in Vietnam.

**Materials and methods:**

From June 2020 to January 2022, a cross-sectional study of patients with acute ischemic stroke was conducted in a tertiary hospital in Vietnam. The dysphagia was evaluated through a bedside screening test using the Gugging Swallowing Screen (GUSS). Factors associated with dysphagia were analysed using univariate and multivariate logistic regression.

**Results:**

The prevalence of dysphagia in patients with acute ischemic stroke was 71.6%, in which the mild, moderate and severe dysphagia accounted for 37.5%, 12.4% and 21.7%, respectively. Dysphagia significantly associated with age group 50–59 (OR = 2.2, 95% CI: 1.2–4.2), age group 60–69 (OR = 1.9, 95% CI: 1.04–3.4), age group >70 (OR = 2.2, 95% CI: 1.2–4.2), brainstem (OR = 4.0, 95% CI: 2.1–7.4), having communication disorder (OR = 1.5, 95% CI: 1.1–7.4) and having facial paralysis (OR = 17.9, 95% CI: 12.0–26.8).

**Conclusion:**

Our study showed that the prevalence of dysphagia is high among patients with acute ischemic stroke in Vietnam. Intervention solutions should focus more on patient groups of higher age group, brainstem stroke, communication disorder and facial paralysis.

## Highlights

• The prevalence of dysphagia in patients with acute ischemic stroke was 71.6%.
• The mild, moderate and severe dysphagia accounted for 37.5%, 12.4% and 21.7%, respectively.
• Dysphagia associated with age group 50–59, age group 60–69, age group >70, brainstem, communication disorder and facial paralysis.

## 1 Introduction

Dysphagia is a symptom of dysfunctional swallowing between the mouth and the stomach [1,2], and it is a common complication in patients with stroke [3,4] and Parkinson's disease [[5], [6], [7]]. A recent meta-analysis reported that 35% of patients with Parkinson's disease developed dysphagia during the course of their disease. The incidence of dysphagia associated with cerebrovascular accidents including stroke ranged from 37% to 78%, according to screening methods with the highest diagnostic sensitivity [8,9]. The consequences of dysphagia are multifaceted, affecting many parts of the human body and causing further mental and physical illnesses. For instance, the presence of dysphagia is shown to worsen the condition of stroke patients, particularly as it is related to an increased risk of pulmonary complications and mortality [10,11]. Dysphagia may also be associated with malnutrition and dehydration in patients with acute stroke [12,13]. According to the American Speech-Language-Hearing Association [14], people with dysphagia experience disinterest, reduced enjoyment, embarrassment, and/or isolation related to eating or drinking.

A meta-analysis in 2016 [15] revealed that risk factors associated with dysphagia included increased age, disease history, and physical frailty, which reduced the ability to carry out activities of daily living. The strongest associations reported in these studies were history of clinical disease, stroke and depression such as in four studies [[16], [17], [18], [19]], followed by frailty and a reduced ability to carry out activities of daily living in three separate studies [17,20,21] and rising age in two studies reporting a significant association of dysphagia with advanced age [22,23].

In recent years, the management and treatment of acute stroke were significantly improved by reperfusion therapy, leading to a significant reduction in mortality [24,25]. Several studies reported that early detection of dysphagia among patients with acute stroke could reduce the risk of complications, length of hospital stay, and cost of treatment [9,26,27]. Although dysphagia is considered an important issue in managing and treating acute stroke, there are currently no studies investigating this issue, especially for patients with acute ischemic stroke in Vietnam. Thus, the purpose of this study was to determine the prevalence of dysphagia and associated factors of dysphagia in patients with acute ischemic stroke in Vietnam.

## 2 Methods

### 2.1 Study setting and patients

This study was conducted at Bach Mai hospital, Vietnam. The study protocol proposal was approved by research ethics committees at Nam Dinh University of Nursing (Document no. 1603/GCN-HDDD). The study registration identifying number (UIN) is researchregistry8203, which is available at https://www.researchregistry.com/. Bach Mai hospital is a tertiary hospital providing health care services to people in Hanoi and the northern provinces of Vietnam. The Neurology Center of Bach Mai hospital is the place to receive and treat patients with neurological diseases, including patients with acute ischemic stroke. Diagnosis of acute ischemic stroke in this center was confirmed by computed tomography (CT) or magnetic resonance imaging (MRI). In this study, the inclusion criteria were patients 18 years of age or older and diagnosed with acute ischemic stroke. In addition, the patients had a Glasgow Coma Scale (GCS) score greater than 13 and were able to communicate. We excluded patients with indications for oxygen or intubation, myocardial infarction, Wernicke's aphasia, dementia, toothlessness, and other medical histories of dysphagia.

### 2.2 Study design and sample size

A cross-sectional study of patients with acute ischemic stroke was conducted between June 2020 and January 2022. The sample size was calculated using the formula for estimating a population proportion with specified absolute precision proposed by the World Health Organization [28]. To determent an appropriate sample size, we used the significance level of 0.05, absolute precision of 0.03 and the anticipated dysphagia prevalence of 73% (based on a pilot study). In addition, we expected a non-response rate of 10% and after cleaning data, a total of 951 patients with acute ischemic stroke were included in the analysis.

### 2.3 Variables

The outcome variable in this study was the dysphagia status of a patient with acute ischemic stroke. The status of dysphagia was defined as whether or not a patient was screened with dysphagia by the Gugging Swallowing Screen (GUSS) [29,30]. Patients were screened by GUSS within 4 h of admission. The GUSS is divided into 2 parts, including the indirect and direct swallowing tests. The highest total GUSS score that a patient can obtain is 20 points, indicating that this patient has normal swallowing ability. Meanwhile, if a patient has a GUSS score of less than or equal to 19, this patient has dysphagia. A lower GUSS score indicates more severe dysphagia. The degree of dysphagia is also divided into 4 levels corresponding to the GUSS scores, namely no dysphagia, mild, moderate and severe dysphagia corresponding to the scores of 20, 15–19, 10–14, and 0–9 points, respectively [29]. Independent variables related to patients included sex (male or female), age groups (<50, 50–59, 60–69 and > 70 years), education (primary school or lower, secondary school and high school or above), obesity (yes or no), site of stroke (hemispheric, brainstem, cerebellar and combined), facial paralysis (yes or no), previous stroke (yes or no), hypertension (yes or no) and diabetes Mellitus (yes or no). In addition, we included a variable of communication disorder (yes or no). The communication disorder was defined as “an impairment in the ability to receive, send, process, and comprehend concepts or verbal, nonverbal and graphic symbol systems” [31]. The status of communication disorder was assessed by a speech-language therapist.

### 2.4 Statistical analysis

Quantitative data were presented as means and standard deviations, whereas qualitative data were presented

as frequencies and percentages. Chi-square tests were used for qualitative variables to compare the difference between groups. Univariate and multivariate logistic regression analysis was conducted to examine factors associated with dysphagia among patients with acute ischemic stroke. All statistical analyses in this study were conducted by STATA® 14. We set the level of statistical significance at P value being less than 0.05. The work has been reported in line with the STROCSS criteria [32].

## 3 Results

A total of 951 consecutive patients with acute ischemic stroke were enrolled in this study. The mean age of patients was 65.3 years (range, 32–90 years). Table 1 presents the general characteristics of patients in the study by different factors. Male patients accounted for nearly two-thirds of the total number of patients. More than two-thirds of the patients were aged 60 years or older, and nearly 40% of the patients had a high school education or higher. Up to 16.4% of the total patients were obese. The majority of patients were diagnosed as hemispheric (78.0%). About 44.2% of patients had communication disorders, while 75.7% had facial paralysis. Only about 19.3% of patients reported having a stroke, 23.4% had diabetes Mellitus, and 67.4% had hypertension.

**Table 1:** Characteristics of patients with acute ischemic stroke in the study.

Variable	N (%) or mean (SD)
Sex	
Male	612 (64.4)
Female	339 (35.6)
Age (year)	65.3 (11.4)
Age group (year)	
<50	93 (9.8)
50-59	176 (18.5)
60-69	333 (35.0)
>70	349 (36.7)
Education	
Primary school or lower	252 (26.6)
Secondary school	325 (34.1)
High school or above	374 (39.3)
Obesity	
No	795 (83.6)
Yes	156 (16.4)
Site of stroke	
Hemispheric	742 (78.0)
Brainstem	148 (15.6)
Cerebellar	21 (2.2)
Combined	40 (4.2)
Communication disorder	
No	531 (55.8)
Yes	420 (44.2)
Facial paralysis	
No	231 (24.3)
Yes	720 (75.7)
Previous stroke	
No	767 (80.7)
Yes	184 (19.3)
Hypertension	
No	310 (32.6)
Yes	640 (67.4)
Diabetes Melitus	
No	728 (76.6)
Yes	223 (23.4)

**Fig. 1:**
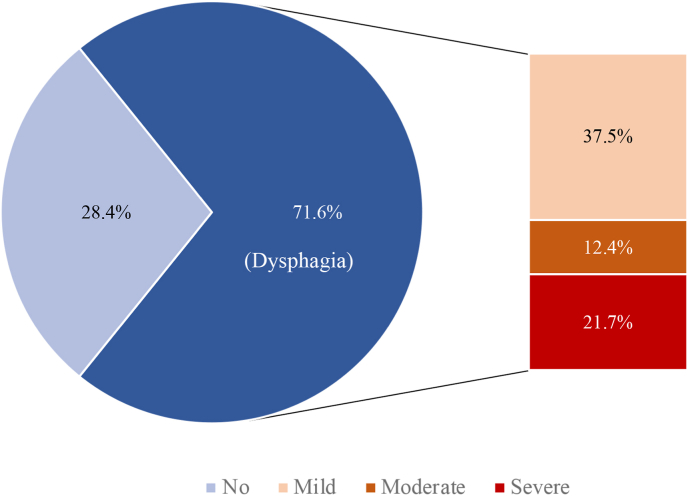
The percentage of dysphagia and the percentage of dysphagia by degree using Gugging Swallowing Screen (GUSS) among patient with acute ischemic stroke.

The overall prevalence of dysphagia in patients with acute ischemic stroke was 71.6%, in which the mild, moderate and severe dysphagia accounted for 37.5%, 12.4% and 21.7%, respectively (Fig. 1). Table 2 shows the prevalence of dysphagia among patients with acute ischemic stroke by various factors. The overall prevalence of dysphagia in patients with acute ischemic stroke was 71.6%, with a statistically significant difference in prevalence between male and female patients (P = 0.04). The prevalence of dysphagia was also significantly different between age groups (P < 0.01) and education levels (P = 0.03). There was a statistically significant difference in the prevalence of dysphagia by the stroke location (P < 0.01). Meanwhile, there was also a statistically significant difference in the prevalence of dysphagia between patients according to communication disorder (P < 0.01) and facial paralysis (P < 0.01) status. Patients with a previous stroke had a statistically significant difference in the prevalence of dysphagia from those who had never had a stroke (P = 0.01).

**Table 2:** Prevalence of dysphagia among patients with acute ischemic stroke by various factors.

Variable	DysphagiaN (%)	P value
All	681 (71.6)	NA
Sex		
Male	256 (75.5)	0.04
Female	425 (69.4)	
Age group		
<50	48 (51.6)	<0.01
5 0-59	117 (66.5)	
60-69	225 (67.6)	
>70	291 (83.4)	
Education		
Primary school or lower	195 (77.4)	0.03
Secondary school	219 (67.4)	
High school or above	267 (71.6)	
Obesity		
No	570 (71.7)	0.89
Yes	111 (71.2)	
Stroke location		
Hemispheric	510 (68.7)	<0.01
Brainstem	130 (87.8)	
Cerebellar	9 (42.9)	
Combined	32 (80.0)	
Communication disorder		
No	350 (65.9)	<0.01
Yes	331 (78.8)	
Facial paralysis		
No	64 (27.7)	<0.01
Yes	617 (85.7)	
Previous stroke		
No	535 (69.8)	0.01
Yes	146 (79.4)	
Hypertension		
No	213 (68.7)	0.16
Yes	468 (73.1)	
Diabetes Mellitus		
No	524 (72.0)	0.65
Yes	157 (70.4)	

Note: NA: Not Applicable.

Table 3 provides factors associated with dysphagia among patients with acute ischemic stroke using univariate and multivariate logistic regression analysis. Dysphagia associated with age group 50–59 (OR = 2.2, 95% CI: 1.2–4.2), age group 60–69 (OR = 1.9, 95% CI: 1.04–3.4), age group >70 (OR = 2.2, 95% CI: 1.2–4.2), brainstem (OR = 4.0, 95% CI: 2.1–7.4), having communication disorder (OR = 1.5, 95% CI: 1.1–7.4) and having facial paralysis (OR = 17.9, 95% CI: 12.0–26.8).

**Table 3:** Factors associated with dysphagia among patients with acute ischemic stroke: Univariate and multivariate logistic regression analysis.

Variable	Dysphagia			
	Crude OR (95% CI)	P value	Adjusted OR (95% CI)	P value
Sex				
Male	1.4 (1.1 - 1.8)	0.047	1.3 (0.9 - 1.9)	0.20
Female	1		1	
Age group				
<50	1		1	
50-59	1.9 (1.1 - 3.1)	0.02	2.2 (1.2 - 4.2)	0.02
60-69	2.0 (1.2 - 3.1)	<0.01	1.9 (1.04 - 3.4)	0.04
>70	4.7 (2.9 - 7.7)	<0.01	5.4 (2.8 - 10.3)	<0.01
Education				
Primary school or lower	1		1	
Secondary school	0.6 (0.4 - 0.9)	<0.01	0.7 (0.5 - 1.2)	0.20
High school or above	0.7 (0.5 - 1.1)	0.10	0.9 (0.6 - 1.6)	0.87
Obesity				
No	1		1	
Yes	1.0 (0.7 - 1.4)	0.89	0.9 (0.5 - 1.4)	0.54
Stroke location				
Hemispheric	1		1	
Brainstem	3.3 (2.0 - 5.5)	<0.01	4.0 (2.1 - 7.4)	<0.01
Cerebellar	0.3 (0.1 - 0.8)	0.02	1.2 (0.4 - 3.8)	0.75
Combined	1.8 (0.8 - 4.0)	0.14	1.8 (0.7 - 4.5)	0.22
Communication disorder				
No	1		1	
Yes	1.9 (1.4 -2.6)	<0.01	1.5 (1.1 - 7.4)	0.02
Facial paralysis				
No	1		1	
Yes	15.6 (11.0 - 22.3)	<0.01	17.9 (12.0 - 26.8)	<0.01
Previous stroke				
No	1		1	
Yes	1.7 (1.1 - 2.5)	0.01	1.5 (0.9 - 2.4)	0.10
Hypertension				
No	1		1	
Yes	1.2 (0.9 -1.7)	0.16	1.0 (0.7 - 1.5)	0.99
Diabetes Mellitus				
No	1		1	
Yes	0.9 (0.7 - 1.3)	0.65	0.8 (0.5 - 1.2)	0.23

Note: OR: Odds Ratio; CI: Confidence Interval.

## 4 Discussion

This is the first study in Vietnam to investigate the prevalence of dysphagia in patients with acute ischemic stroke. We found that the prevalence of dysphagia was high among patients with acute ischemic stroke. In addition, we have discovered several important implicated factors for dysphagia in these patients. These findings inform both clinical and public health decision making about dysphagia intervention for patients with acute ischemic stroke, and also further research in the field.

Although we found a high prevalence of dysphagia in patients with acute ischemic stroke, this prevalence was still within the range reported by previous studies [4,[33], [34], [35], [36]]. In comparison with Asian communities, such as Koreans (32.3%) [37], Chinese (39.4%) [38], and Japanese (58.7–73.7%) [39], our data lie within the upper limit. The high prevalence of dysphagia in our study could be explained that patients admitted to geriatric hospitals due to stroke or other health problems were more likely to have this condition, and the condition is also commonly more severe. However, our neurology center is a leading center in the region, so most acute strokes in the region are referred to us, thus including all levels of stroke condition. In addition, the use of different diagnostic methods may also influence the prevalence of dysphagia. In this study, we used GUSS, and it proved to be a useful method with high reliability [29]. Moreover, we had a large sample size and continuously recruited patients for more than 2 years. Therefore, we believe that the patients we included in the study could represent the population with acute ischemic stroke in the north of Vietnam.

In this study, we found that age groups were associated with dysphagia, which was also reported in previous studies [[40], [41], [42]]. As age increases, swallowing function tends to be negatively affected [40,43,44]. Therefore, when an elderly patient has a stroke, there is an increased risk of negatively impacting swallowing function. Given the possible consequences for patients with dysphagia, it is necessary to pay attention and caution in screening elderly patients with acute ischemic stroke, especially those over the age of 70.

In this study, we also found that stroke location was associated with dysphagia, but only for brainstem strokes. Several previous studies showed that stroke location is one of the predictors of dysphagia and prolonged dysphagia after stroke. However, the study by Marcel et al. reported that stroke location was not associated with dysphagia [45]. Our sample size was larger than that of Marcel et al.'s study [45], increasing a power to detect an association between dysphagia and brainstem strokes. This relationship may need further research to confirm, so it could help to prevent the later consequences for the patients with acute ischemic strokes who had dysphagia.

Several studies showed that the previous stroke was a factor associated with dysphagia [46,47]. This association was detected only in univariate logistic regression analysis in our study. However, previous stroke and dysphagia had no association in the multivariate logistic regression analysis. The results of our study were inconsistent with previous studies [46,47], possibly due to differences in the system for recording stroke information. Collecting information about the previous stroke from patients or their family members might have potential biases to study results. In addition, we found an association between dysphagia and communication disorder and facial paralysis. Communication disorder and facial paralysis may be predictors of diagnosing the dysphagia status in patients with acute ischemic strokes.

We found that dysphagia prevalence was significantly different between men and women in this study. However, dysphagia only had a statistically significant association in univariate logistic regression analysis. We did not find an association between dysphagia in the multivariable logistic regression analysis. An association between dysphagia and sex was reported in several previous studies, but these studies only included elderly patients [[48], [49], [50]]. The inconsistency between our study and these studies may be due to the difference in study participants. While, the results of our study are similar to Kawashima et al.'s in that they also did not find an association between dysphagia and sex [51]. Thus, this issue should still be studied further to confirm the existence of this association.

No association was found between obesity and dysphagia in patients with acute ischemic strokes in this study; this was similar to the results of a previous study [52]. In addition, we did not find a significant association between dysphagia and hypertension, and between dysphagia and diabetes. It is possible that hypertension and diabetes were common diseases in the study population, especially in the elderly, thus preventing an association between hypertension and these diseases. However, hypertension and dysphagia were found to have a statistically significant association in the previous study [52]. We believe that more studies are needed to clarify this relationship.

We would like to highlight here some limitations of this study. First, the current classification of dysphagia was from mild to severe dysphagia, so it may overestimate the prevalence of dysphagia. In addition, because the specificity of GUSS is low [30], there might be bias in estimating the prevalence of dysphagia. Furthermore, in this study, we only screened the dysphagia status of patients who had suffered from acute ischemic stroke without information on the previous history of dysphagia or swallowing impairment. This is a group of patients of advanced age, so it is possible that a certain proportion of patients now suffer from swallowing impairment before having an acute ischemic stroke. In addition, certain patients with indications for oxygen or intubation, myocardial infarction, Wernicke's aphasia, dementia, toothlessness, and other medical histories of dysphagia were excluded from the study, which might underestimate the prevalence of dysphagia. Finally, because this was a cross-sectional study design, caution should be exercised with interpreting the results related to the cause-effect relationship.

## 5 Conclusion

The study highlights the common prevalence of dysphagia among patients with stroke. Given that dysphagia is associated with higher age group, brainstem stroke, communication disorder and facial paralysis, we recommend that intervention programs focus on patients being older with brainstem stroke. Clinical algorithm becomes more complex as it requires attention to associated conditions such as communication disorders and facial paralysis, especially the cause of stroke.

## Availability of data and materials

The datasets used and/or analysed during the current study are available from the corresponding author on reasonable request.

## Ethical approval

The study protocol proposal was approved by research ethics committees at Nam Dinh University of Nursing (Document no. 1603/GCN-HDDD)

## Sources of funding

No funding was received in this study.

## Authors' contributions

NTTH, THT, LTT and VDK developed the study concept and design, analysed and interpreted the data, and wrote the manuscript. VHK, DTTH and TTT contributed to the data collection and improvement of the manuscript. NVH contributed to improve the manuscript. All authors read and approved the final manuscript.

## Registration of research studies

1. Name of the registry: researchregistry.com
2. Unique Identifying number or registration ID: researchregistry8203
3. Hyperlink to your specific registration (must be publicly accessible and will be checked):https://www.researchregistry.com/register-now#home/registrationdetails/62f9b8ed7e507500222ad4d2/

## Guarantor

Nguyen Thi Thu Hien.

## Provenance and peer review

Not commissioned, externally peer-reviewed.

## Declaration of competing interest

The authors declare that they have no competing interests.

## List of Abbreviations

CI: Confidence Interval
CT: Computed tomography
GCS: Glasgow Coma Scale
GUSS: Gugging Swallowing Screen
MRI: Magnetic Resonance Imaging
OR: Odds Ratio
